# A commentary on “Preemptive left stellate ganglion block reduces the incidence and severity of cardiac surgery-associated acute kidney injury: a randomized clinical trial”

**DOI:** 10.1097/JS9.0000000000003179

**Published:** 2025-08-21

**Authors:** Fu-Shan Xue, Dan-Feng Wang, Xiao-Chun Zheng

**Affiliations:** a Department of Anesthesiology, Shengli Clinical Medical College of Fujian Medical University, Fuzhou University Affiliated Provincial Hospital, Fujian Provincial Hospital, Fuzhou, China; b Department of Anesthesiology, Beijing Anzhen Hospital, Capital Medical University, Beijing, China

*Dear Editor*,

By conducting a single-center, randomized controlled trial with a total of 138 patients who underwent elective cardiac surgery under cardiopulmonary bypass, Zhou et al^[1]^. demonstrated that preemptive single-injection left stellate ganglion block before surgery significantly decreased the incidence and severity of cardiac surgery-associated acute kidney injury (CSA-AKI). Given that CSA-AKI can complicate postoperative recovery and may place patients at increased risks of in-hospital death and chronic kidney disease development, their findings have important implication. Many aspects of this research have been well done, especially both the intention-to-treat and per-protocol principles are conducted for data analysis. However, as a randomized controlled trial to determine the effect of a studied intervention on the occurrence and severity of CSA-AKI, all of other factors that may influence the assessment of primary outcomes must be standardized for avoidance of potential biases. We noted that several issues of this study were not well addressed and would have affected the interpretation of the results.

First, the participants were American Society of Anesthesiologists physical status classifications 3 and 4, with a mean age of more than 64 years. In the baseline characteristics of participants, Zhou et al^[1]^. did not provide preoperative hemoglobin and serum protein levels. Furthermore, it was unclear if the two groups were comparable with respect to the incidences of preoperative anemia and hypoalbuminemia. Available evidence indicates that preoperative anemia and hypoalbuminemia are causally attributable to the development of CSA-AKI^[2,3]^. In addition, this study excluded the participants with preoperative abnormal renal function by serum creatinine (SCr) > 133 μM^9^ and the two groups had normal preoperative SCr levels. It must be noted that SCr is not the gold standard for determining preoperative abnormal renal dysfunction. It is reported that preoperative occult renal dysfunction with an estimated glomerular filtration rate of 60-89 mL/min/1.73 m^2^ is common among patients with normal preoperative SCr for cardiac surgery, with a prevalence of 44.1% in patients aged less than 65 years^[4]^. Especially, preoperative occult renal dysfunction has been independently associated with the occurrence of severe CSA-AKI and progression of chronic kidney disease after cardiac surgery^[4,5]^.

Second, according to flow diagram of the trial protocol provided in Zhou et al.’ article^[1]^, 5 participants in each groups were not included in the per-protocol analysis due to a poor-quality ultrasonographic imaging of renal blood flow. This seems not to be in line with the principles of per-protocol analysis because these participants had received the studied interventions based on randomized assignment, and the resistive index and pulsatility index of the left renal artery by ultrasonography were not primary outcomes of this study. Most important, in the statistical analysis, Zhou et al^[1]^. described that multiple imputation with Fully Conditional Specification method was used to address the missing data. However, it was unclear which outcome variables were missed and treated by this method for the intention-to-treat and per-protocol analyses.

Third, CSA-AKI within 7 days postoperatively was defined according to the 2012 guidelines of Kidney Disease: Improving Global Outcomes. Zhou et al^[1]^. did not specify whether the SCr levels used for diagnosis of CSA-AKI had been adjusted based on postoperative fluid balance, though a positive fluid balance can dilute SCr levels and the SCr-adjustment for fluid balance can significantly improve the diagnosis of CSA-AKI^[6,7]^.

Fourth, postoperative recovery outcomes including the extubation time, duration of ICU stay and length of hospitalization were significantly improved in the stellate ganglion block group. However, Zhou et al^[1]^. did not observe the duration of CSA-AKI, even if it is directly proportional to short- and long-term outcomes of patients^[8]^. Especially, Zhou et al^[1]^. did also not state if the two groups were comparable in terms of postoperative management data and complication occurrences. In available literatures, postoperative vasopressor exposure, anemia and transfusion, hyperglycemia, hypovolemia, low cardiac output syndrome and infection have been identified as the independent risk factors of CSA-AKI^[8,9]^.

We argue that clarifying the above issues would improve the transparency of this research design and help the interpretation of main findings. Furthermore, we ensure that our article is compliant with the TITAN Guidelines 2025, as shown in TITAN Guideline Checklist 2025^[10]^.

## Data Availability

All data is available.

## Research registration unique identifying number

No necessary.

## References

[1] Zhou W Yu Y Tian S. Preemptive left stellate ganglion block reduces the incidence and severity of cardiac surgery-associated acute kidney injury: a randomized clinical trial. Int J Surg 2025. doi:10.1097/JS9.0000000000002913 PMC12626528 40643258

[2] Lin YW Wang Q Lu PS. Early acute kidney injury recovery in elderly patients undergoing valve replacement surgery. J Cardiothorac Vasc Anesth 2024;38:2261–68. 39019743 10.1053/j.jvca.2024.06.027

[3] Wiedermann CJ Wiedermann W Joannidis M. Causal relationship between hypoalbuminemia and acute kidney injury. World J Nephrol 2017;6:176–87. 28729966 10.5527/wjn.v6.i4.176 PMC5500455

[4] Xu J Yu J Xu X. Preoperative hidden renal dysfunction add an age dependent risk of progressive chronic kidney disease after cardiac surgery. J Cardiothorac Surg 2019;14:151. 31438993 10.1186/s13019-019-0977-9 PMC6704689

[5] Wijeysundera DN Karkouti K Beattie WS Rao V Ivanov J. Improving the identification of patients at risk of postoperative renal failure after cardiac surgery. Anesthesiology 2006;104:65–72. 16394692 10.1097/00000542-200601000-00012

[6] Moore E Tobin A Reid D Santamaria J Paul E Bellomo R. The impact of fluid balance on the detection, classification and outcome of acute kidney injury after cardiac surgery. J Cardiothorac Vasc Anesth 2015;29:1229–35. 26005020 10.1053/j.jvca.2015.02.004

[7] Jin J Xu J Xu S. Hemodilution is associated with underestimation of serum creatinine in cardiac surgery patients: a retrospective analysis. BMC Cardiovasc Disord 2021;21:61. 33517880 10.1186/s12872-021-01879-w PMC7849106

[8] Romagnoli S Ricci Z Ronco C. Perioperative acute kidney injury: prevention, early recognition, and supportive measures. Nephron 2018;140:105–10. 29945154 10.1159/000490500

[9] Jiang Y Song Y. Analysis of risk factors and intervention strategies for acute kidney injury after cardiac valve replacement. J Inflamm Res 2023;16:3523–29. 37614810 10.2147/JIR.S425485 PMC10443686

[10] Agha RA Mathew G Rashid R. Transparency In The reporting of Artificial INtelligence-the TITAN guideline. Prem J Sci 2025;10:100082.

